# Continental Reference Point: Harmonized Human Biomonitoring across Europe

**DOI:** 10.1289/ehp.123-A71

**Published:** 2015-03-01

**Authors:** Kris S. Freeman

**Affiliations:** Kris S. Freeman has written for the National Institutes of Health and the National Park Service.

The Consortium to Perform Human Biomonitoring on a European Scale (COPHES) was launched to collect population-representative data on environmental exposures for the European Union (EU) as a whole. In this issue of *EHP*, investigators describe results from DEMOCOPHES, the demonstration project that proved the feasibility of a harmonized approach across 17 EU nations.^1^ “We have shown that working together on a common protocol allows us to obtain comparable results on an EU-wide level,” says lead author Greet Schoeters, program manager of environment and health at VITO, the Flemish Institute for Technological Research.

This type of human biomonitoring can be used to track exposure trends and potential impacts of environmental regulations. For example, ongoing biomonitoring by the German Environmental Survey (GerES) suggests that a 1992 recommendation against the use of amalgam fillings in children and women of child-bearing age may have contributed to decreasing urinary mercury levels in that country’s population.^2^

**Figure:**
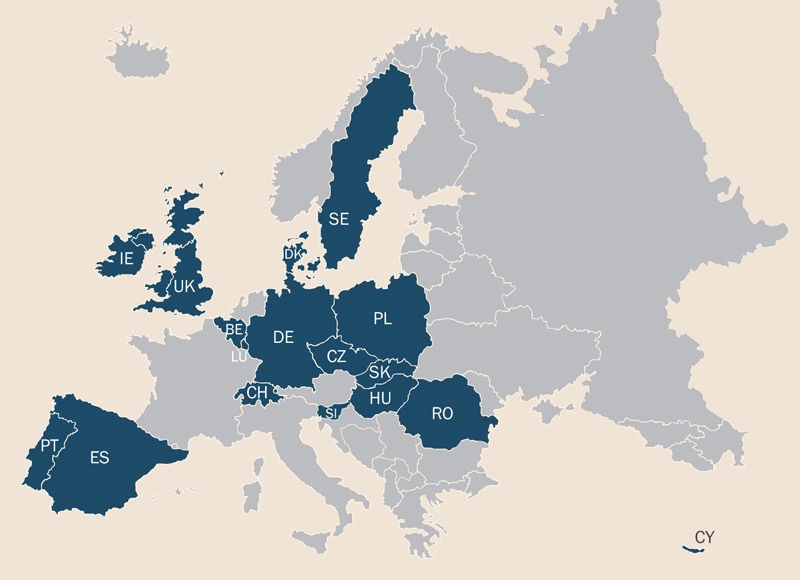
A new study has demonstrated that human biomonitoring can be conducted in a harmonized fashion across the European Union. © Shutterstock; Joseph Tart/EHP

For the current study, researchers analyzed hair and urine samples from 1,844 mother–child pairs recruited from 17 countries. Half the participants lived in rural areas, and half lived in urban areas. All were healthy with no known metabolic disturbances. Samples yielded information on exposures to mercury, cadmium, nicotine, and several phthalates found in personal care products and food packaging. To help ensure validity and transferability of results, researchers developed harmonized protocols and quality controls for chemical and data analyses.

As with the U.S. National Health and Nutrition Examination Survey, exposures were associated with known lifestyle and diet predictors, and biomarkers for children closely matched those of their mothers. “This overall pattern provides confidence that the effort to validly collect and analyze these samples and companion exposure information was successful,” says Lesa Aylward, a toxicologist at Summit Toxicology LLP, who was not involved with the study.

The vast majority of biomarker concentrations were below health-based guidance values set by the Joint FAO/WHO Expert Committee on Food Additives (JECFA) and the German Human Biomonitoring Commission, as well as published values from independent scientists. Eastern, southern, and (to a lesser extent) western European countries clustered together based on similar biomarker patterns, differences that Schoeters says are probably related to lifestyle factors, environmental conditions, and possibly regulation.

Levels of metabolites of di(2-ethylhexyl) phthalate (DEHP) indicated ubiquitous exposure to this compound even though its use is restricted in the EU.^3^^,^^4^^,^^5^ Regular consumption of chewing gum and ice cream was associated with higher average concentrations of multiple phthalate metabolites among children and mothers.^1^ These treats “may serve as a proxy for other factors, such as levels of processed food in the diet,” says Schoeters.

For monoisobutyl phthalate, average European values in both mothers and children were 3–4 times higher than those observed in the United States. Compared with the United States, Europeans on average had lower biomarker concentrations of monobenzyl phthalate and monoethyl phthalate, and higher concentrations of mono-*n*-butyl phthalate and DEHP metabolites, but these differences were less dramatic.^1^

Hair mercury concentrations increased with reported consumption of fish and shellfish. Concentrations were 35% higher for children and 30% for mothers living in urban areas, the only result in this study that showed differences between residents of urban and rural areas. Hair mercury was highest among children living in Spain and Portugal, whose average concentrations were 6 and 7 times higher, respectively, than the European average. The proportion of participants whose mercury levels exceeded the JECFA/WHO provisional threshold value differed considerably by country, with no participants exceeding the threshold in most northern and central European countries and up to 33% of mothers exceeding the threshold in countries with high fish consumption.^1^

Concentrations of cotinine (a metabolite of nicotine) varied widely by country, with levels lowest in countries with the strongest anti-smoking legislation.^6^ Smoking was also associated with increased cadmium levels, which were 30% higher among mothers who smoked.^1^ Smoking is an important source of cadmium exposure because tobacco plants preferentially accumulate cadmium from the soil.^7^

“The study is a step towards European reference values,” says coauthor Ludwine Casteleyn, a researcher at the University of Leuven Center for Human Genetics in Belgium. “Currently European countries, together with the European Commission, are exploring the possibilities for a more sustainable system that should be able to support environmental health policy, at a European and a national level.”
